# A Simple Technique to Manage Gag Reflex

**DOI:** 10.7759/cureus.35403

**Published:** 2023-02-24

**Authors:** Shreya Colvenkar, Bhuvaneshwari Kalmath, Vishnu Priya Cherukuri, Jayasri Vanapalli, Sri Varsha Tirukovalur

**Affiliations:** 1 Department of Prosthodontics, MNR Dental College and Hospital, Sangareddy, IND; 2 Department of Oral Medicine and Radiology, MNR Dental College and Hospital, Sangareddy, IND; 3 Department of Orthodontics, MNR Dental College and Hospital, Sangareddy, IND

**Keywords:** dentistry. gagging, gagging, eye massager, anxiety, tray, impression

## Abstract

Gagging poses a clinical difficulty to the dentist in all facets of therapy, from diagnostic techniques to active treatment. Various treatment methods to manage the gagging include behavioral techniques, acupressure, acupuncture, hypnosis, systemic desensitization, and pharmacological techniques. This article presents a straightforward method for getting rid of the gag reflex using an eye massager. Eye massagers can help reduce the stress and anxiety associated with dental procedures by providing an effective alternative to traditional methods. This technique can be a valuable aid in making short dental procedures more comfortable and successful.

## Introduction

The gag reflex is one of the primary challenges that must be managed during prosthodontic treatments, especially when making an impression. Though gagging has a multifactorial etiology, an over-reactive gag reflex can be caused by anxiety in a few people [1]. This anxiety, ranging from mild to severe, can make the entire dental experience unpleasant and stressful.

Various factors that cause gag reflex can be classified into local, medical, social, psychological, iatrogenic, and dental.

Local factors include a deviated nasal septum, nasal obstruction, postnasal drip, and sinusitis. Medical factors include chronic gastritis, carcinoma of the stomach and pancreas, and partial gastrectomies. Social factors include heavy smoking due to hypersensitivity, coughing, chronic catarrh, and chronic alcoholism. Psychological factors include stress, phobias, alcoholism, and fear. Iatrogenic factors include water and suction tubes, instrumentation, local anesthesia, and radiography. Dental factors include poor retention, the surface finish of dentures, an inadequate posterior palatal seal, restricted tongue space in dentures, and overextended borders.

Strategies such as education, proper communication, and modifying treatment plans are valuable ways to reduce this fear and help ensure successful outcomes. Fear of visits to the dentist often affects a patient's willingness to seek care, and many patients experience gag reflexes due to the mere sight of a local anesthetic injection or impression tray.

There are several methods that have been suggested to help manage this reflex, such as acupressure [2], acupuncture [3], hypnosis [4], a virtual reality headset [5], an eye mask with music [6], desensitization exercises [4], and cognitive-behavioral therapy [7]. The diagnosis of the cause, not only the symptoms, is necessary for proper care. The patient should be evaluated individually, and an attempt should be made to identify the situations that cause gagging. A thorough history should be taken, with empathic, specific, and open questions. A clinical examination with a ball burnisher should then be performed to identify trigger points.

This article describes a simple remedy for suppressing the gag reflex using an eye massager.

## Technical report

**Figure 1:**
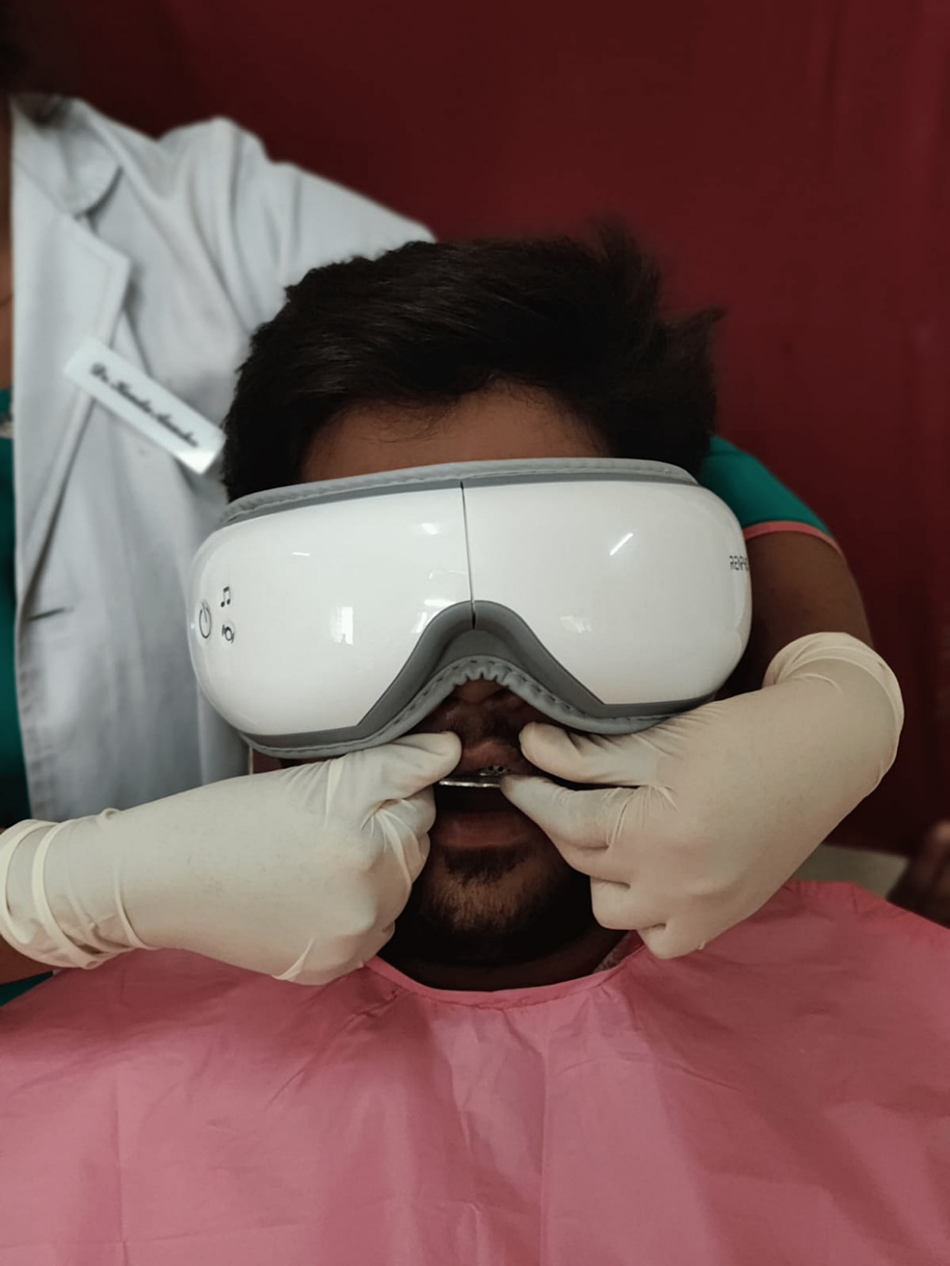
Patient with an eye massager during the impression-making process

Establish a positive rapport with patients by instilling trust in the dental treatment. Explain briefly the treatment procedure that will be used. Instruct the patient to wear an eye massager and activate the mode of his choice, such as vibration, heat massage, or plain massage. Request that the patient listens to music to relax. Describe how doing so will help people feel less anxious and avoid the gag reflex (Figure 1).

Mix the impression material according to the manufacturer’s instructions, load it onto the impression tray, and insert it into the patient’s mouth until it sets.

## Discussion

Dental procedures can be a daunting experience for patients, especially those with mild gag reflexes [1]. Depending on severity, gagging can be classified as very mild, mild, moderate, severe, or very severe. Mild gagging can be controlled by the patient or dentist by applying simple measures. Various management techniques have been mentioned in the literature, but the key to management lies in understanding the cause and treating it accordingly [2-5]. This will help to better execute the procedure to achieve positive and gag-free results. It is incumbent upon the treating dentist to calm the patient so he or she can carry out the treatment with ease. Thus, it is important that every dental clinician have a thorough knowledge of gagging for its skillful management and for better execution of the procedures to attain positive and gag-free results.

Eye massagers can help reduce the stress and anxiety associated with dental procedures by providing a calm and stress-free environment. The eye massager comes with a built-in heat massager and a vibrator that provides the patient with a relaxing and calm environment. It also includes a built-in speaker to listen to music during the massage. It temporarily diverts the patient’s sight from a distressing environment, thus allowing the patient to carry out the treatment with ease.

It makes short dental procedures like making impressions, injecting local anesthesia [8], or taking intraoral radiographs more comfortable for the patient. This technique is valuable for patients with mild gag reflexes, as it helps them remain calm and relaxed during the procedure. For patients with a severe, disruptive gag reflex, when used in combination with other techniques, the headset can be an effective tool. In order to ensure the safety of patients, headsets must be appropriately spaced and disinfected between appointments. The cost of headsets varies from $15 to $50, making them an affordable solution for dental practices. The simple time-saving method helps reduce stress and anxiety associated with dental procedures while still providing quality care to their patients, thus improving patient satisfaction. This technique, tried on a couple of anxious patients, successfully prevented a mild gag reflex during impression-making. The large size of the headset may present a small challenge for the clinician when making impressions. Further research needs to be carried out to understand how this method is better compared to other techniques in the management of mild gag reflexes.

## Conclusions

The gag reflex presents a major obstacle for both dentists and patients when it comes to the successful completion of treatment. Eye massagers can help prevent the anxiety associated with dental procedures by providing an effective alternative to traditional methods. Through an audiovisual aid, the patient can easily unwind and get over their fear of the tray being inserted during the impression-making process.

## References

[1] Conny DJ, Tedesco LA (1983). The gagging problem in prosthodontic treatment. Part I: description and causes. J Prosthet Dent.

[2] Vachiramon A, Wang WC (2002). Acupressure technique to control gag reflex during maxillary impression procedures. J Prosthet Dent.

[3] Fiske J, Dickinson C (2001). The role of acupuncture in controlling the gagging reflex using a review of ten cases. Br Dent J.

[4] Morse DR, Hancock RR, Cohen BB (1984). In vivo desensitization using meditation-hypnosis in the treatment of tactile-induced gagging in a dental patient. Int J Psychosom.

[5] Colvenkar S, Ali MM (2022). Management of gag reflex with a virtual reality headset. J Prosthet Dent.

[6] Colvenkar S, Reddy V, Thotapalli S, Deepa Rani K, Bharadwaj S (2022). A simple step-by-step technique for the management of gagging in edentulous patient. Cureus.

[7] Neumann JK, McCarty GA (2001). Behavioral approaches to reduce hypersensitive gag response. J Prosthet Dent.

[8] Kunusoth R, Colvenkar S, Alwala AM, Sampreethi S, Ahmed MS (2022). Massage therapy to control anxiety before extraction of an impacted tooth. Cureus.

